# Evaluating the Effects of Kinesthetic Biofeedback Delivered Using Reaction Wheels on Standing Balance

**DOI:** 10.1155/2018/7892020

**Published:** 2018-06-11

**Authors:** Muhammad Raheel Afzal, Amre Eizad, Carlos Ernesto Palo Peña, Jungwon Yoon

**Affiliations:** ^1^School of Integrated Technology, Gwangju Institute of Science and Technology, Gwangju 61005, Republic of Korea; ^2^School of Mechanical and Aerospace Engineering, Gyeongsang National University, Jinju 52828, Republic of Korea

## Abstract

Aging, injury, or ailments can contribute to impaired balance control and increase the risk of falling. Provision of light touch augments the sense of balance and can thus reduce the amount of body sway. In this study, a wearable reaction wheel-based system is used to deliver light touch-based balance biofeedback on the subject's back. The system can sense torso tilt and, using reaction wheels, generates light touch. A group of 7 healthy young individuals performed balance tasks under 12 trial combinations based on two conditions each of standing stance and surface types and three of biofeedback device status. Torso tilt data, collected from a waist-mounted smartphone during all the trials, were analyzed to determine the efficacy of the system. Provision of biofeedback by the device significantly reduced RMS of mediolateral (ML) trunk tilt (*p* < 0.05) and ML trunk acceleration (*p* < 0.05). Repeated measures ANOVA revealed significant interaction between stance and surface on reduction in RMS of ML trunk tilt, AP trunk tilt, ML trunk acceleration, and AP trunk acceleration. The device shows promise for further applications such as virtual reality interaction and gait rehabilitation.

## 1. Introduction

Standing with a stable posture is a capability that most of us take for granted and so considered to be a simple task. The reality, on the contrary, is totally opposite to this assumption. The achievement of the stable standing posture is possible through a synergetic collaboration of various faculties of the human body. The mechanism for maintaining postural stability can be divided into three parts: sensing, processing, and actuation. The sense of balance is achieved by the utilization of the vestibular system, visual input from the eyes, and proprioceptive input from the lower extremities [1]. The communication and processing of all the sensor data are carried out by the central nervous system (CNS), which generates the actuation signals according to those data, which are also communicated by the CNS. The actuation signals are implemented by the musculoskeletal system [2]. A weakness, injury, or disorder of any of these systems involved may hamper the execution of the balance maintaining task, leading to postural instability [3]. The faculties involved may be weak due to congenital disorders or degraded due to aging, disease, or injury, thus causing postural instability. Reduced postural control, apart from causing lack of confidence and reduced independence, may also be the cause of falling, thereby causing injury [4]. Therefore, remedial measures need to be taken to improve postural control.

The remedial measures include the implementation of various rehabilitation strategies [5]. Rehabilitation strategies include exercises or tasks that enhance posture control and are tailored according to the particular patient and modified according to their progress [6]. The task performance is sometimes accompanied by the use of various assistive devices such as orthotics, systems that induce a particular pattern of movement and biofeedback systems [7]. It is in the realm of these devices that modern engineering technology is now being extensively applied [8–10]. The inclusion of automated systems in the rehabilitation process can reduce therapist involvement, allowing them to provide service to a greater number of patients. Compact and cost efficient systems may even allow the user to use them and perform rehabilitation tasks in the comfort of their own home, increasing the chance of the patients adhering to the prescribed exercises till the desired level of rehabilitation has been achieved [11].

One group of rehabilitation devices that is being explored for automated rehabilitation is the biofeedback generation devices [12]. These devices provide feedback to the user according to their performance. This feedback is in a form that can be perceived by the user using one or more of their senses. The different modes of feedback commonly exploited for balance rehabilitation are visual [13–16], audio [17–19], and haptic [20–22]. The haptic feedback is further divided into tactile and kinesthetic feedback. These modes may also be used in conjunction with each other to form a multimodal system. Such multimodal systems, with one system previously developed by us [23], have also shown positive outcomes with regards to balance rehabilitation [24]. The visual cue systems require display devices which make the overall system cumbersome and inappropriate for use as a wearable device. The audio-based system is more compact, but it utilizes the sense of hearing which is already being utilized by the user for listening to the therapist's commands and other environmental sounds. Thus, the haptic-based systems are most suited for unobtrusive delivery of biofeedback.

In the field of kinesthetic haptic biofeedback, one point of great interest is the concept of “light touch.” Light touch refers to a fingertip contact with a rigid surface that involves forces which are not strong enough to give mechanical support to the person but are strong enough to be perceived by the somatosensory system. This very low force stimulus when processed by the CNS acts to augment the proprioceptive input coming to the brain and can thus make up for the weakened balance sensing capability. Light touch is known to improve postural control [25–27]. We have previously developed a system that utilized light-touch biofeedback delivered to the hand by a Phantom Omni® device for balance training [28]. We have also devised a multimodal biofeedback generation system for balance training by combining light-touch biofeedback from the Phantom Omni device with a visual biofeedback provision system [23, 29]. Experiments with both these systems produced promising results, but the biggest limitation in both cases was that the systems were not portable. A further variation of light touch is interpersonal light touch. This refers to very low force generating contact of parts of the body with another person or a static or moving object. Interpersonal light touch is not just limited to fingertip contact but also encompasses touch at other parts of the body. It has been seen that interpersonal light touch also acts to reduce body sway [30]. Johannsen et al. have shown that, under some test conditions, interpersonal light touch to the shoulder yields better results than fingertip contact during performance of balancing tasks [31]. Krishnamoorthy et al. have shown that light-touch interface of a fixed device with the neck and head has a more profound effect in improving the postural stability than that with the finger [32]. Therefore, further exploration of interpersonal light touch, administered to a part of the body other than the finger, such as the subject's back, as a balance rehabilitation tool is warranted. Furthermore, to the best of authors' knowledge, a wearable system for inducing light touch on a subject's back without the involvement of another person has not yet been evaluated. A wearable system, by virtue of its wearability, is usually easier to use and less cumbersome than fixed or portable systems. Therefore, exploration of the possibility to apply currently available technology to devise a wearable, controlled light touch-inducing system is also warranted. Such a wearable system may benefit rehabilitation of subjects with balance impairment. A wearable haptic biofeedback system may also be used at home by individuals who find it difficult to go to a clinic for therapy sessions.

The system presented in this paper utilizes the concept of interpersonal light touch by inducing forces on the user's back to give them feedback of their body sway. It is a wearable system where instead of using a stationery object or another person to generate the reaction forces, we have utilized reaction wheels (RWs). The torso tilt is sensed using an on-board inertial measurement unit (IMU), and the data are processed by an on-board microcontroller which then actuates four RWs to generate the torque required to induce the light touch. In this paper, we have evaluated the effect of these cues as a balance biofeedback on young healthy subjects performing various standing tasks.

## 2. Materials and Methods

### 2.1. System Description

**Figure 1:**
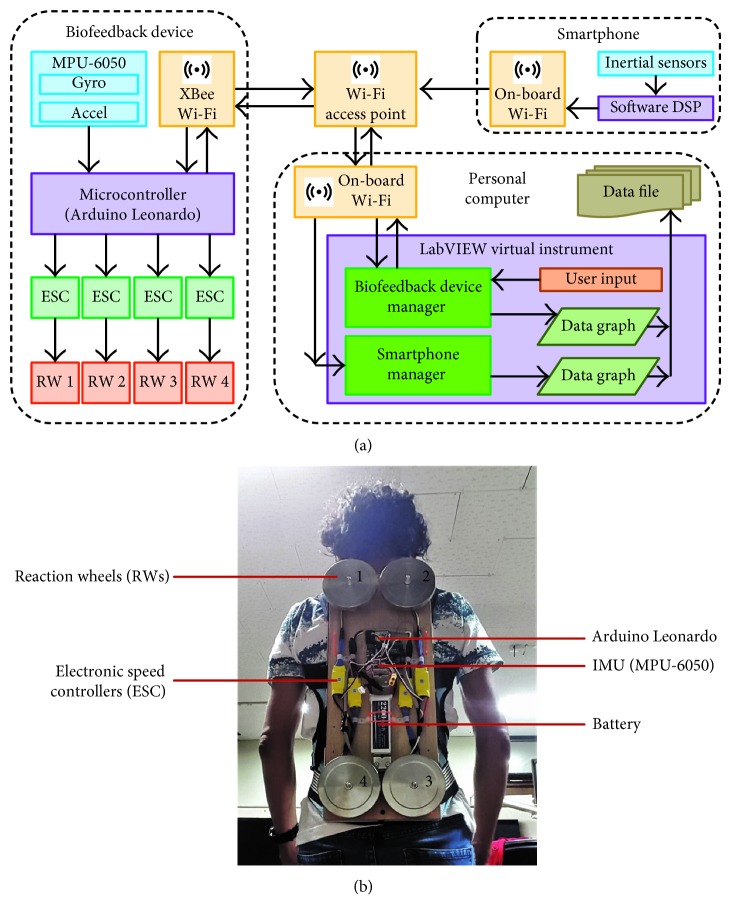
System Design. (a) Block diagram of the experimental setup with indication of data flows. The smartphone is not part of the biofeedback system and is used for data collection purposes only. The biofeedback device can function on its own, and the PC is only required for an initial configuration of the device and for data monitoring and logging. (b) Developed hardware of the biofeedback device as worn by a participant during testing.

We have devised a rather simple, easy-to-use system that consists of a wearable RW-based biofeedback generation device and a PC-based system for device configuration and viewing and logging of sensor data [33]. Although the biofeedback device is designed to function as a stand-alone device without being connected to the PC, during experimental trials, it is connected to the PC for monitoring purposes. A smartphone-based torso tilt sensing module is also used during experimental trials; it is not part of the biofeedback system and is only used to gather experimental data. The biofeedback device and smartphone both communicate with the PC over a Wi-Fi link to allow completely wireless operation. The feedback device has an on-board IMU which it uses to sense any changes in its orientation. The values read for the IMU are communicated to the microcontroller where they are processed to determine torso tilt in the mediolateral (ML) plane. Based on this calculated tilt angle, the microcontroller generates control signals for the RW motors. The data are also communicated to the PC where it is stored for if further processing is desired. The body sway values measured by the smartphone are also communicated to the PC where they are stored for any further analysis. The block diagram of the complete experimental setup with indication of data flow is shown in Figure 1(a).

The feedback device generates intuitive balance cues in the form of light touch generated due to the induction of torque from the RW. The device is composed of four RWs attached to an easy-to-wear harness. The wheel motors are connected to electronic speed controllers (HW25\30A ESC) which allow the on-board Arduino microcontroller (Arduino Leonardo by DFRobot) to control the motors. The microcontroller takes sensory input from the IMU (MPU-6050 by Invensense). A lithium polymer battery is used as the power source for this module, and communication is handled using an XBee Wi-Fi transceiver. The biofeedback device prototype as worn by a participant with labeled parts is shown in Figure 1(b).

**Figure 2:**
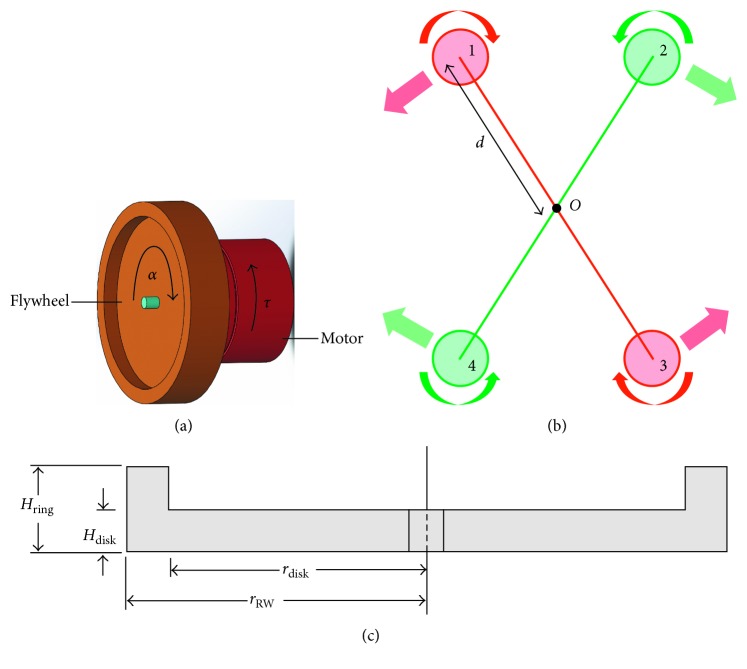
(a) Torque production in a reaction wheel assembly due to angular acceleration. (b) Layout of the RW in the biofeedback device with dimensional labels. (c) Cross section of the biofeedback device flywheel with dimensional labels.

The RWs are usually used in spacecraft for attitude control, but their compact design makes them ideal for use in applications where low magnitude torque is required. Every RW used in our system consists of a brushless DC motor (A2212/13T, 1000 KV) attached to a high-inertia flywheel. When this flywheel is accelerated or decelerated, a reaction torque is induced on the motor [34]. A simple representation of this phenomenon is shown in Figure 2(a).

The reaction torque induced on the motor is, by virtue of the motor's connection with the mounting harness, converted into a linear force that is perceived by the user as light touch. The device is worn by the user like a backpack with two shoulder straps and one strap at the waist. The torque generated by the RW manifests itself as forces acting on the contact areas of these straps with the user's body. These forces, instead of being felt at individual points, provide a total sensation of lightly trying to tilt the user's body to the right-hand or left-hand side.

**Figure 3:**
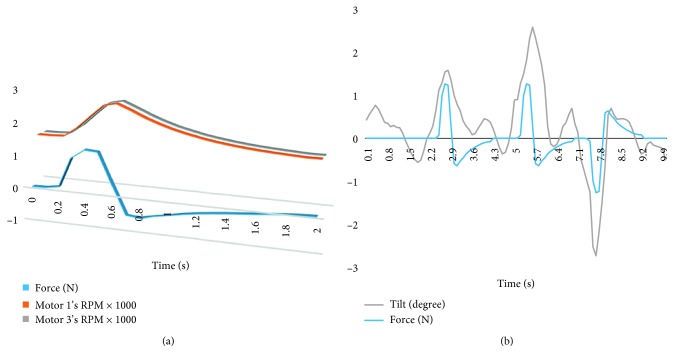
(a) Representative plot of motor 1 and motor 3 RPM and the consequently generated force. (b) The force generated by the system in relation to the torso tilt.

Our feedback device consists of 4 RWs arranged in an “X”-shaped configuration. The RW pairs located at the ends of each diagonal work in tandem to generate torque in one direction, so the “X”-shaped configuration allows for the generation of torques in two directions. The arrangement of RW with accompanying dimensional tags is shown in Figure 2(b). The touch forces generated by these torques correspond to mediolateral (ML) trunk tilt in both the right and left directions [33]. When the user's torso tilt towards their right-hand side exceeds the set threshold, leftward-directed cue is generated, and vice versa. The cue itself consists of a singular impulse application of force every time the torso tilt exceeds the set threshold. The amount of torque experienced by the motor is dependent on the amount of acceleration taking place and the moment of inertia of the RW (Figure 3(a)). Moment of inertia of a rotating body is the measure of its mass and its distance from the axis of rotation. The moment of inertia for the wheel used in this research is calculated using (1). The variables used in this equation are defined in Figure 2(c). Since the device is designed to be wearable, its size is limited, and thus, the size of the components used is also restricted. Therefore, the selected wheel diameter is 10 cm:

(1) $$\begin{array}{c} I_{\text{RW}}=\rho \frac{\pi }{2}\left[H_{\text{ring}}\left(r_{\text{RW}}^{4}-r_{\text{disk}}^{4}\right)+H_{\text{disk}}r_{\text{disk}}^{4}\right]. \end{array}$$

Using (1), the moment of inertia of each of the RW used in this research is *I*_RW_=1.625 × 10^−4^ kgm^2^.

The separation distance between the centers of the wheels is *d* = 19.5 cm. The total mass moment of inertia of the RW array about its center was found using the parallel axis theorem, which yielded the following equation:

(2) $$\begin{array}{c} I_{\text{tot}}=4\left(I_{\text{RW}}+m_{\text{W}}d^{2}\right), \end{array}$$

where *d* is the distance of the wheel center from the center of rotation of the device and *m*_W_ is the mass of the wheel found using the following equation:

(3) $$\begin{array}{c} m_{\text{W}}=\rho \pi \left[r_{\text{RW}}^{2}h_{\text{ring}}-r_{\text{disk}}^{2}\left(h_{\text{ring}}-h_{\text{disk}}\right)\right]. \end{array}$$

The mass of each RW was found to be *m*_W_=0.130 kg. Therefore, from (2), the total mass moment of inertia of the complete RW array is *I*_tot_=0.0204 kgm^2^.The RWs were empirically tested in couples to validate the design. The maximum force generated by each RW couple was 1.24 N and can thus be considered as light touch. The device has a total weight of 4.20 kg inclusive of all its components.  Figure 3(b) shows the net force being generated by the system in relation to the torso tilt.

### 2.2. Experimental Setup

The PC-based module of the system runs the LabVIEW environment in which an application is developed to receive data from the biofeedback device and the smartphone tilt sensor for display and logging and allows the operator to configure the biofeedback device. The program utilizes bidirectional UDP communication over Wi-Fi to communicate with the devices. It can receive sensor data from the feedback device and can be used to switch the RW motors on and off and to control their speed. Communication with the smartphone is unidirectional; the PC only receives body sway data from the module. The smartphone used in this research is a Pantech Vega IM-A850L that has a quad-core 1.5 GHz CPU with 2 GB of RAM and runs the Android® operating system. We have previously utilized the smartphone as a reliable body sway assessment tool during both stance and gait conditions [23, 28, 29, 35–37]. The smartphone runs an application that measures the trunk tilt in terms of the ML and anteroposterior (AP) angles and sends these data via UDP over Wi-Fi to the PC.

Seven healthy young participants took part in the experimental study and performed prescribed balance tasks to check the effectiveness of biofeedback provided by our system. The details of these participants are given in Table 1. None of the subjects had any history of sensorimotor disorders. This study was conducted according to the Declaration of Helsinki and had ethical approval from the Institutional Review Board at the Gwangju Institute of Science and Technology. All subjects gave written informed consent prior to data collection.

The subjects were asked to wear the feedback device and the smartphone in order to conduct the experimental tests. The feedback device is provided with straps so that it can be worn like a backpack, and the smartphone is attached to the waist with the help of an elastic belt. For the purpose of conducting the experiments presented here, the cue generation threshold for trunk tilt was set at ±1° about the vertical. With all the hardware in place and configuration completed, the subjects were asked to maintain their balance while standing with two distinct stance postures on two distinct surfaces for 30 seconds each. The data obtained during first and last 5 seconds of each trial were not utilized during analysis. The prescribed stance postures were tandem-Romberg and single-leg. In tandem-Romberg stance, one foot is placed in front of the other with heel of the anterior foot touching the toe of the posterior foot, and the nondominant leg is in the posterior position. In single-leg stance, the subject stands on the nondominant leg with the contralateral limb held in approximately 20° of hip flexion, 45° of knee flexion, and neutral position in the frontal plane. Subject's kicking preference is used to determine leg dominance. The surfaces used in these tests were solid ground and a platform made of foam. The platform was used to simulate soft ground conditions. It had the dimensions of 600 × 600 × 150 mm and was made using high resilience foam that had a density of 48 kg/m^3^ and tensile strength of 83 kPa. The subjects were explained appropriate utilization of kinesthetic biofeedback for balance control prior to start of the experimental trials. The biofeedback device worn by the subjects delivered light-touch balance cues. The subjects, utilizing these cues, tried to achieve the objective of balancing themselves in the prescribed stance on the designated surface. The stance conditions and surface conditions would enable us to identify the efficacy of the proposed system when operated under different conditions. We anticipated that the system will have greater efficacy when the user is performing balance tasks in relatively more unstable conditions.

Each participant performed balancing tasks under a total of twelve trial combinations composed of three conditions of the biofeedback device, two ground conditions, and two distinct standing postures (3 × 2 × 2 = 12). The three biofeedback device conditions were as follows: not wearing the device, wearing the device but it is not providing any biofeedback, and wearing the device while it is providing biofeedback. The participants stood on normal ground for stable support and on a foam platform that simulated unstable ground conditions. The two standing postures assumed by the participants were the tandem-Romberg stance and the one-leg stance.

Balancing trials under the mentioned 12 different sets of conditions were carried out with all the conditions being applied to all the participants in a random order. The abbreviations associated with the testing conditions are tabulated in Table 2. “N” refers to trials done without wearing the biofeedback device. “O” refers to trials done while wearing the device but it is not switched on. “B” refers to trials done while wearing the device and it is switched on and providing biofeedback.

### 2.3. Data Collection and Analysis

Body sway is a meaningful indicator that can be used to recognize the balance of a human being during upright standing posture [38]. The smartphone attached to the subjects' waist measured trunk tilt angles during trials and communicated them wirelessly to the PC. The ML and AP, trunk tilt, and acceleration data were recorded on the PC. In postexperimental analysis, RMS values of ML trunk tilt, AP trunk tilt, ML trunk acceleration, and AP trunk acceleration were calculated. Afterwards, we carried out statistical analysis of the recorded data to make detailed observations about balance performance [39]. Using dependent *t*-test, we compared the body sway under N and O conditions with statistical significance defined as *p* < 0.05. A 3-way repeated measures ANOVA was conducted (factors: feedback (O, B), stance (T, S), and surface (G, F)) for analysis of the trunk tilt and acceleration parameters. In addition, we calculated reduction in RMS values of ML trunk tilt (RMS-ML-tilt-R), AP trunk tilt (RMS-AP-tilt-R), ML trunk acceleration (RMS-ML-acceleration-R), and AP trunk acceleration (RMS-AP-acceleration-R) by calculating absolute difference between O and B conditions. A 2-way repeated measures ANOVA was conducted to investigate the effects of stance (factor) and surface (factor) on reduction of RMS values of ML trunk tilt, AP trunk tilt, ML trunk acceleration, and AP trunk acceleration. Post hoc multiple comparison tests were conducted using the Bonferroni correction method.

## 3. Results and Discussion

Mean ± standard deviation (SD) for all subjects' RMS of ML trunk tilt, RMS of AP trunk tilt, RMS of ML trunk acceleration, and RMS of AP trunk acceleration are shown in Table 3. Results of the *t*-tests showed that there was no statistically significant difference between N (without wearing the biofeedback device) and O (wearing the device, but it is not switched on) for the dependent variables (ML trunk tilt, AP trunk tilt, ML trunk acceleration, and AP trunk acceleration) in all trial conditions (Table 4). This shows that wearing the device did not significantly affect the users' balance.

Table 5 shows the statistics of the 3-way repeated measures ANOVA with factors: feedback, stance, and surface. Results of the 3-way repeated measures ANOVA revealed that all main effects and interactions were significant for RMS of ML trunk tilt. Post hoc analysis revealed significant difference of RMS of ML trunk tilt at all levels of each factor and between factors. However, RMS of AP trunk tilt exhibited significant main effects and interactions of the stance and surface factors. The post hoc analysis revealed significant difference of RMS of AP trunk tilt at all levels of surface and stance factors and between these factors. RMS of ML trunk acceleration exhibited significant main effects of feedback, stance, and surface and interaction of the stance and surface. Post hoc analysis revealed significant difference of RMS of ML trunk acceleration at all levels of each factor and between factors. RMS of AP trunk acceleration exhibited significant main effects and interaction of the stance and surface factors. The post hoc analysis revealed a significant difference of RMS of AP trunk acceleration at all levels of surface and stance factors and between these factors.

**Figure 4:**
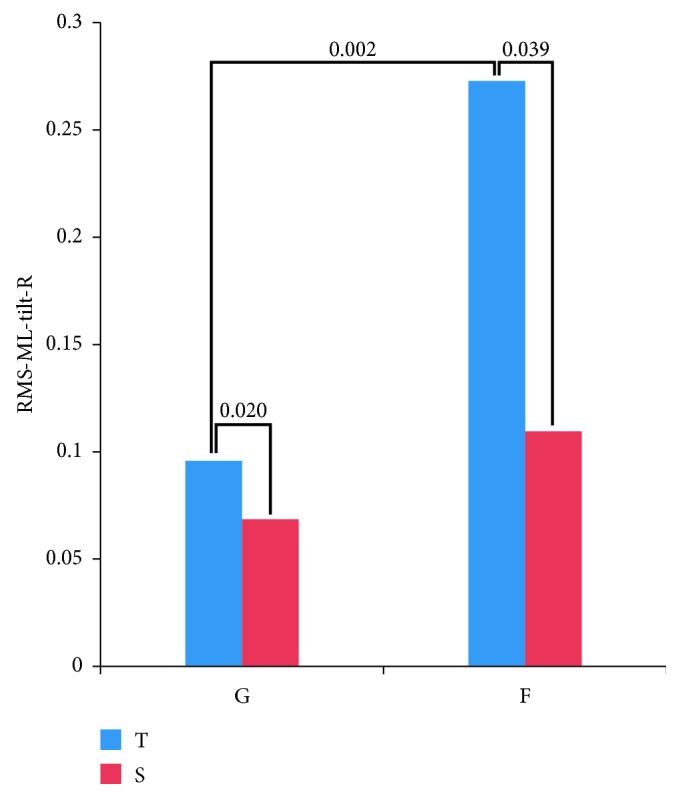
Result of post hoc simple main effects analysis following the 2-way repeated measures ANOVA. Reduction in RMS of ML trunk tilt is compared for the different stance and surface. The bars represent the mean reduction in RMS of ML trunk tilt.

Results of the 2-way repeated measures ANOVA revealed that both main effects and the interaction were insignificant for RMS-ML-acceleration-R, RMS-AP-tilt-R, and RMS-AP-acceleration-R. However, main effects of stance (*p* value = 0.012), surface (*p* value = 0.003), and stance × surface interaction (*p* value = 0.005) were statistically significant for RMS-ML-tilt-R. Due to significant interaction, post hoc analysis was conducted to evaluate simple main effects for RMS-ML-tilt-R (Figure 4). Statistically significant difference was found in reduction of RMS values of ML trunk tilt between tandem-Romberg and single-leg stance on ground (*p* value = 0.020) and on foam (*p* value = 0.039) surfaces. Statistically significant difference was also found in reduction of RMS values of ML trunk tilt between ground and foam conditions in tandem-Romberg stance (*p*=0.002). However, no statistically significant difference in reduction of RMS values of ML trunk tilt was found between ground and foam conditions in single-leg stance. From this result, we can observe that reduction in RMS of ML trunk tilt was more on the foam surface relative to the ground as expected. However, when comparing stance conditions, the reduction in RMS of ML trunk tilt was more in tandem-Romberg stance in comparison to the single-leg stance. This outcome can be attributed to the deficiency of AP directional cues/assistance from the system.

In this paper, the effect of provision of kinesthetic biofeedback on the subject's back for balance is presented. When the means and standard deviations (SD) of the data collected during experimental trials are observed, it shows that provision of biofeedback delivered by our system reduced body sway in the participants. This may be attributed to the somatosensory augmentation provided by the haptic biofeedback. Somatosensory augmentation is known to improve standing stability [40]. In order to evaluate the effects of the biofeedback device, RMS of ML and AP trunk tilt, and RMS of ML and AP trunk acceleration are observed. The device currently provides balance cues in the ML direction upon trunk sway in the ML direction. Therefore, the results obtained in the ML direction are the focus of this research. RMS of ML trunk tilt has been shown to be a reliable marker of postural control in multiple prior studies [41–43]. Likewise, RMS of ML trunk acceleration has been shown to be a reliable measure of judging balance during standing trials [44–46]. During the study, the participants exhibited no significant differences in RMS of ML trunk tilt and RMS of ML trunk acceleration, between not wearing the device (N) and wearing the device with no feedback conditions (O). This shows that the wearing of the device did not affect the postural stability of the participants. On the contrary, while comparing RMS of ML trunk tilt under no feedback (O) and biofeedback (B) conditions, statistically significant differences were found in all stance and surface conditions. Similar results for RMS of ML trunk acceleration were observed. Hence, kinesthetic biofeedback generated by our system had a significant effect on the postural stability of the subjects. This is in line with our hypothesis that application of light-touch cues to a subject's back works to reduce their body sway. 2-way repeated measures ANOVA revealed significant interaction between stance and surface on reduction in RMS of ML trunk tilt between no device and biofeedback conditions. This indicates that the amount of postural stability improvement varies in relation to the stance and the surface conditions. In contrast, there was no significant interaction between stance and surface on reduction in RMS of ML trunk acceleration, RMS of AP trunk tilt, and RMS of AP trunk acceleration between no device and biofeedback conditions.

The system generates a force magnitude of 1.24 N which can be considered as light touch-based biofeedback and is sufficient for standing balance tasks as evidenced in previous related works [26, 27]. It might be necessary to increase the force magnitude in order to apply this biofeedback method during locomotor tasks as previous related works dealing with light-touch feedback during walking have utilized up to 4 N force [47, 48]. A number of recent studies have reported on the promising effects of vibrotactile biofeedback on standing balance [49, 50]. Thus, it is a point of interest to study this system for provision of kinesthetic biofeedback in comparison with a vibrotactile balance biofeedback system to determine the differences in their performance. A study to compare the neurophysiological effects that these systems may have on a particular set of users is also envisioned.

A limitation of this study is the small number of participants, but several other published works related to effect of biofeedback devices have also reported trials with small sample size [51, 52]. Through the testing carried out during this research, we not only were able to judge the effects of the device on performance of prescribed balance tasks but also were able to uncover some shortcomings of the current device prototype. The participants were able to wear the device with ease, but they were not comfortable with its weight. They were in general view that wearing the device for extended period of time will become uncomfortable due to its weight. Reduction in weight of the system is thus a point of consideration for our future work. This may be possible through variations in material selection so that the system may become suitable for application in extended rehabilitation schemes. One such scheme is the use of haptic biofeedback devices in conjunction with virtual reality to enhance poststroke balance and mobility [53]. As the system provides balance cues in the ML direction only, to enhance its capabilities, there is a need to make it a two-dimensional cue delivery system that can provide cues in both the ML and AP directions. In order to do this, the appropriate layout of reaction wheels needs to be determined and hardware needs to be developed that can generate the desired forces without being too heavy. The current study didn't identify the effects of added weight of the system on the balance recovery of the users during large sway. In future work, we will also observe the postural control of the users in detail under condition of postural perturbations.

## 4. Conclusions

A wearable biofeedback device which generates light-touch biofeedback in correspondence to torso movement in the ML directions is evaluated in this research. The tests were conducted with participants without any balance impairments, and imbalance was induced by the use of imbalance inducing standing stances and an unstable standing surface. The level of balance achieved by the participants was judged based on their body sway in ML and AP. The outcomes observed during initial trials with healthy young subjects point towards an important addition to the balance training procedures. We observed that our method of delivering kinesthetic biofeedback can be applied to balance rehabilitation through the use of specifically designed balance tasks. Being wearable, the system has high potential for use at home or in outpatient clinics for balance training exercises. Experimental trials conducted with young healthy subjects supported the feasibility of the system as a balance training aid. In future prototype, the system should be designed to minimize the current limitations. We plan to use this system to perform long-term balance training of individuals with upright balance issues. Furthermore, exploiting the wearable nature of the system, we also plan to explore the benefits of utilizing this device as a balance assistance aid during gait.

## Figures and Tables

**Table 1:** Details of the young healthy subjects who participated in the study.

Participants	Age (year)	Height (cm)	Weight (kg)	Gender	
7	27 ± 3	161 ± 9	70 ± 6.5	Male = 6	Female = 1

**Table 2:** A summary representation of all the testing conditions used in the experimental trials along with their related abbreviation tags.

Stance condition	Surface condition					
	Ground (G)			Foam (F)		
Tandem-Romberg (T)	NTG	OTG	BTG	NTF	OTF	BTF
Single-leg (S)	NSG	OSG	BSG	NSF	OSF	BSF

**Table 3:** RMS values of the measured parameters.

Parameter	Stance	Surface					
		Ground (G)			Foam (F)		
		Device					
		N	O	B	N	O	B
ML trunk tilt (degree)	Tandem-Romberg (T)	0.5192 ± 0.0381	0.4961 ± 0.0223	0.4234 ± 0.0102	1.3747 ± 0.0668	1.3724 ± 0.1051	1.1019 ± 0.0564
	Single-leg (S)	1.1251 ± 0.0450	1.1287 ± 0.0257	1.0582 ± 0.0124	2.1152 ± 0.0391	2.0980 ± 0.0256	2.0058 ± 0.0411

AP trunk tilt (degree)	Tandem-Romberg (T)	1.0172 ± 0.0374	1.0219 ± 0.0470	1.0328 ± 0.2759	1.9030 ± 0.0552	1.9048 ± 0.0574	1.8932 ± 0.0501
	Single-leg (S)	1.6114 ± 0.0287	1.6205 ± 0.0238	1.6274 ± 0.0275	2.4284 ± 0.0368	2.4318 ± 0.0446	2.4104 ± 0.0249

ML trunk acceleration (m/s^2^)	Tandem-Romberg (T)	0.0635 ± 0.0025	0.0629 ± 0.0029	0.0514 ± 0.0018	0.1062 ± 0.0026	0.1054 ± 0.0037	0.0926 ± 0.0043
	Single-leg (S)	0.0956 ± 0.0016	0.0946 ± 0.0034	0.0858 ± 0.0031	0.1459 ± 0.0032	0.1451 ± 0.0036	0.1348 ± 0.0049

AP trunk acceleration (m/s^2^)	Tandem-Romberg (T)	0.0614 ± 0.0196	0.0619 ± 0.0012	0.0620 ± 0.0011	0.1082 ± 0.0029	0.1075 ± 0.0038	0.1079 ± 0.0034
	Single-leg (S)	0.1063 ± 0.0027	0.1070 ± 0.0038	0.1066 ± 0.0031	0.1621 ± 0.0023	0.1613 ± 0.0028	0.1618 ± 0.0019

**Table 4:** Comparison of N and O trial conditions with *t*-test (*p* value).

Parameter	Stance condition	Surface condition	
		Ground (G)	Foam (F)
ML trunk tilt	Tandem-Romberg (T)	0.084	0.950
	Single-leg (S)	0.779	0.103

AP trunk tilt	Tandem-Romberg (T)	0.825	0.847
	Single-leg (S)	0.299	0.817

ML trunk acceleration	Tandem-Romberg (T)	0.310	0.390
	Single-leg (S)	0.351	0.547

AP trunk acceleration	Tandem-Romberg (T)	0.541	0.278
	Single-leg (S)	0.498	0.180

**Table 5:** ANOVA statistics of the dependent variables.

Effect	Parameter			
	ML trunk tilt	AP trunk tilt	ML trunk acceleration	AP trunk acceleration
Feedback	*F*(1, 6) = 86.645, *p* < 0.001	*F*(1, 6) = 0.497, *p*=0.507	*F*(1, 6) = 104.653, *p* < 0.001	*F*(1, 6) = 0.165, *p*=0.699

Stance	*F*(1, 6) = 2368.311, *p* < 0.001	*F*(1, 6) = 2120.584, *p* < 0.001	*F*(1, 6) = 1500.545, *p* < 0.001	*F*(1, 6) = 2900.932, *p* < 0.001

Surface	*F*(1, 6) = 5434.270, *p* < 0.001	*F*(1, 6) = 2305.391, *p* < 0.001	*F*(1, 6) = 4238.458, *p* < 0.001	*F*(1, 6) = 2899.990, *p* < 0.001

Feedback × stance	*F*(1, 6) = 10.457, *p*=0.018	*F*(1, 6) = 0.181, *p*=0.686	*F*(1, 6) = 2.500, *p*=0.165	*F*(1, 6) = 0.062, *p*=0.811

Feedback × surface	*F*(1, 6) = 23.599, *p*=0.003	*F*(1, 6) = 2.351, *p*=0.176	*F*(1, 6) = 0.600, *p*=0.468	*F*(1, 6) = 0.628, *p*=0.458

Stance × surface	*F*(1, 6) = 42.320, *p*=0.001	*F*(1, 6) = 13.773, *p*=0.010	*F*(1, 6) = 7.235, *p*=0.036	*F*(1, 6) = 26.467, *p*=0.002

Feedback × surface × stance	*F*(1, 6) = 18.800, *p*=0.005	*F*(1, 6) = 0.129, 0.732	*F*(1, 6) = 0.001, *p*=0.981	*F*(1, 6) = 0.043, *p*=0.842

## Data Availability

Data supporting the conclusions of this research are included within the article.

## References

[1] Nashner L. M. (1981). Analysis of stance posture in humans. *Motor Coordination*.

[2] Hassan B. S., Mockett S., Doherty M. (2001). Static postural sway, proprioception, and maximal voluntary quadriceps contraction in patients with knee osteoarthritis and normal control subjects. *Annals of the Rheumatic Diseases*.

[3] Sackley C. M. (1991). Falls, sway, and symmetry of weight-bearing after stroke. *International Disability Studies*.

[4] Herdman S. J., Schubert M. C., Tusa R. J. (2001). Strategies for balance rehabilitation. *Annals of the New York Academy of Sciences*.

[5] Badke M. B., Shea T. A., Miedaner J. A., Grove C. R. (2004). Outcomes after rehabilitation for adults with balance dysfunction. *Archives of Physical Medicine and Rehabilitation*.

[6] Alsalaheen B. A., Whitney S. L., Mucha A., Morris L. O., Furman J. M., Sparto P. J. (2013). Exercise prescription patterns in patients treated with vestibular rehabilitation after concussion. *Physiotherapy Research International*.

[7] Hesse S. (2003). Rehabilitation of gait after stroke: evaluation, principles of therapy, novel treatment approaches, and assistive devices. *Topics in Geriatric Rehabilitation*.

[8] Ahamed N. U., Sundaraj K., Ahmad R. B., Rahman S. M. (2012). Biosensors assisted automated rehabilitation systems: a systematic review. *International Journal of the Physical Sciences*.

[9] Patel S., Park H., Bonato P., Chan L., Rodgers M. (2012). A review of wearable sensors and systems with application in rehabilitation. *Journal of Neuroengineering and Rehabilitation*.

[10] Ahamed N. U., Sundaraj K., Ahmad R. B., Nadarajah S., Shi P. T., Rahman S. M. (2011). Recent survey of automated rehabilitation systems using EMG biosensors. *Journal of Physical Therapy Science*.

[11] Forkan R., Pumper B., Smyth N., Wirkkala H., Ciol M. A., Shumway-Cook A. (2006). Exercise adherence following physical therapy intervention in older adults with impaired balance. *Physical Therapy*.

[12] Huang H., Wolf S. L., He J. (2006). Recent developments in biofeedback for neuromotor rehabilitation. *Journal of Neuroengineering and Rehabilitation*.

[13] Bayouk J. F., Boucher J. P., Leroux A. (2006). Balance training following stroke: effects of task-oriented exercises with and without altered sensory input. *International Journal of Rehabilitation Research*.

[14] Lange B., Flynn S., Proffitt R., Chang C. Y., “Skip”Rizzo A. (2010). Development of an interactive game-based rehabilitation tool for dynamic balance training. *Topics in Stroke Rehabilitation*.

[15] Srivastava A., Taly A. B., Gupta A., Kumar S., Murali T. (2009). Post-stroke balance training: role of force platform with visual feedback technique. *Journal of the Neurological Sciences*.

[16] Young W., Ferguson S., Brault S., Craig C. (2011). Assessing and training standing balance in older adults: a novel approach using the ‘Nintendo Wii’ Balance Board. *Gait and Posture*.

[17] Franco C., Fleury A., Gumery P. Y., Diot B., Demongeot J., Vuillerme N. (2013). iBalance-ABF: a smartphone-based audio-biofeedback balance system. *IEEE Transactions Biomedical Engineering*.

[18] Dozza M., Horak F. B., Chiari L. (2007). Auditory biofeedback substitutes for loss of sensory information in maintaining stance. *Experimental Brain Research*.

[19] Chiari L., Dozza M., Cappello A., Horak F. B., Macellari V., Giansanti D. (2005). Audio-biofeedback for balance improvement: an accelerometry-based system. *IEEE Transactions on Biomedical Engineering*.

[20] Afzal M. R., Jan Y., Hassan S., Yoon J., Lee J., Lee M. C., Liu H., Ryu J. H. (2013). Analysis of active haptic feedback effects on standing stability. *Intelligent Robotics and Applications*.

[21] Sienko K. H., Vichare V. V., Balkwill M. D., Wall C. (2010). Assessment of vibrotactile feedback on postural stability during pseudorandom multidirectional platform motion. *IEEE Transactions on Biomedical Engineering*.

[22] Lee B. C., Kim J., Chen S., Sienko K. H. (2012). Cell phone based balance trainer. *Journal of Neuroengineering and Rehabilitation*.

[23] Afzal M. R., Oh M. K., Choi H. Y., Yoon J. (2016). A novel balance training system using multimodal biofeedback. *Biomedical Engineering Online*.

[24] Bechly K. E., Carender W. J., Myles J. D., Sienko K. H. (2013). Determining the preferred modality for real-time biofeedback during balance training. *Gait and Posture*.

[25] Kouzaki M., Masani K. (2008). Reduced postural sway during quiet standing by light touch is due to finger tactile feedback but not mechanical support. *Experimental Brain Research*.

[26] Jeka J. J., Lackner J. R. (1994). Fingertip contact influences human postural control. *Experimental Brain Research*.

[27] Jeka J. J. (1997). Light touch contact as a balance aid. *Physical Therapy*.

[28] Afzal M. R., Byun H. Y., Oh M. K., Yoon J. (2015). Effects of kinesthetic haptic feedback on standing stability of young healthy subjects and stroke patients. *Journal of Neuroengineering and Rehabilitation*.

[29] Afzal M. R., Oh M. K., Yoon J. Development of a multimodal biofeedback system for balance training.

[30] Johannsen L., Guzman-Garcia A., Wing A. M. (2009). Interpersonal light touch assists balance in the elderly. *Journal of Motor Behavior*.

[31] Johannsen L., Wing A. M., Hatzitaki V. (2012). Contrasting effects of finger and shoulder interpersonal light touch on standing balance. *Journal of Neurophysiology*.

[32] Krishnamoorthy V., Slijper H., Latash M. L. (2002). Effects of different types of light touch on postural sway. *Experimental Brain Research*.

[33] Afzal M. R., Palo Peña C. E., Yoon J. Development of a wearable device based on reaction wheels to deliver kinesthetic cues for balance training.

[34] Sidi M. J. (1997). *Spacecraft Dynamics and Control: A Practical Engineering Approach*.

[35] Afzal M. R., Oh M. K., Lee C. H., Park Y. S., Yoon J. (2015). A portable gait asymmetry rehabilitation system for individuals with stroke using a vibrotactile feedback. *BioMed Research International*.

[36] Afzal M. R., Pyo S., Oh M. K., Park Y. S., Lee B. C., Yoon J. Haptic based gait rehabilitation system for stroke patients.

[37] Afzal M. R., Pyo S., Oh M. K., Park Y. S., Yoon J. Identifying the effects of using integrated haptic feedback for gait rehabilitation of stroke patients.

[38] Baloh R. W., Corona S., Jacobson K. M., Enrietto J. A., Bell T. (1998). A prospective study of posturography in normal older people. *Journal of the American Geriatrics Society*.

[39] Afzal M. R., Pyo S., Oh M. K., Park Y. S., Yoon J. (2018). Evaluating the effects of delivering integrated kinesthetic and tactile cues to individuals with unilateral hemiparetic stroke during overground walking. *Journal of Neuroengineering and Rehabilitation*.

[40] Shull P. B., Damian D. D. (2015). Haptic wearables as sensory replacement, sensory augmentation and trainer–a review. *Journal of Neuroengineering and Rehabilitation*.

[41] Sienko K. H., Balkwill M. D., Oddsson L. I., Wall C. (2013). The effect of vibrotactile feedback on postural sway during locomotor activities. *Journal of Neuroengineering and Rehabilitation*.

[42] Wall C. (2010). Application of vibrotactile feedback of body motion to improve rehabilitation in individuals with imbalance. *Journal of Neurologic Physical Therapy*.

[43] Horak F. B., Dozza M., Peterka R., Chiari L., Wall C. (2009). Vibrotactile biofeedback improves tandem gait in patients with unilateral vestibular loss. *Annals of the New York Academy of Sciences*.

[44] Moe-Nilssen R. (1998). Test-retest reliability of trunk accelerometry during standing and walking. *Archives of Physical Medicine and Rehabilitation*.

[45] Whitney S. L., Roche J. L., Marchetti G. F. (2011). A comparison of accelerometry and center of pressure measures during computerized dynamic posturography: a measure of balance. *Gait and Posture*.

[46] Mancini M., Horak F. B., Zampieri C., Carlson-Kuhta P., Nutt J. G., Chiari L. (2011). Trunk accelerometry reveals postural instability in untreated Parkinson’s disease. *Parkinsonism and Related Disorders*.

[47] Boonsinsukh R., Panichareon L., Phansuwan-Pujito P. (2009). Light touch cue through a cane improves pelvic stability during walking in stroke. *Archives of Physical Medicine and Rehabilitation*.

[48] Boonsinsukh R., Panichareon L., Saengsirisuwan V., Phansuwan-Pujito P. (2011). Clinical identification for the use of light touch cues with a cane in gait rehabilitation poststroke. *Topics in Stroke Rehabilitation*.

[49] Lee B. C., Fung A., Thrasher T. A. (2018). The effects of coding schemes on vibrotactile biofeedback for dynamic balance training in Parkinson’s disease and healthy elderly individuals. *IEEE Transactions on Neural Systems and Rehabilitation Engineering*.

[50] Bao T., Carender W. J., Kinnaird C. (2018). Effects of long-term balance training with vibrotactile sensory augmentation among community-dwelling healthy older adults: a randomized preliminary study. *Journal of Neuroengineering and Rehabilitation*.

[51] Ma C. Z., Zheng Y. P., Lee W. C. (2018). Changes in gait and plantar foot loading upon using vibrotactile wearable biofeedback system in patients with stroke. *Topics in Stroke Rehabilitation*.

[52] Alahakone A. U., Senanayake S. A. (2010). A real-time system with assistive feedback for postural control in rehabilitation. *IEEE/ASME Transactions on Mechatronics*.

[53] Fung J., Perez C. F. Sensorimotor enhancement with a mixed reality system for balance and mobility rehabilitation.

